# Murphy’s Law and Ophthalmic Complications in a Patient With Type 1 Diabetes Mellitus

**DOI:** 10.7759/cureus.40584

**Published:** 2023-06-18

**Authors:** Zineb Barkhane, Maria Qureshi, Ahmed Jamil, Purnashree Chowdhury, Muhammad Kamran, Mohamedalamin Alnoor Altayb Ismail, Shahzeb Saeed

**Affiliations:** 1 Faculté de Médecine et de Pharmacie, Université Hassan II de Casablanca, Casablanca, MAR; 2 Family Medicine, Ayub Medical College, Abbottabad, PAK; 3 Internal Medicine, Mayo Hospital Lahore Pakistan, Lahore, PAK; 4 Neurology, Comilla Medical College Hospital, Chittagong, BGD; 5 Internal Medicine, Mayo Hospital, Lahore, PAK; 6 Internal Medicine, Ibrahim Malik Teaching Hospital, Khartoum, SDN; 7 Internal Medicine, Army Medical College Rawalpindi, Islamabad, PAK

**Keywords:** anti -vegf, neovascular glaucoma, tass, ophthalmology, diabete type 1

## Abstract

Diabetes can lead to various acute clinical complications, although the occurrence of ophthalmic signs and symptoms is uncommon. Neovascular glaucoma (NG), a rare complication associated with diabetes mellitus, is one such condition. Additionally, anti-vascular endothelial growth factor (VEGF)-induced toxic anterior segment syndrome (TASS) is a rare complication of intravitreal bevacizumab.

In this case report, we present a unique case of a patient with juvenile diabetes (type 1 diabetes mellitus) who presented to the emergency room (ER) with typical features of diabetic ketoacidosis (DKA) accompanied by bilateral ocular pain. Subsequent investigation revealed secondary angle-closure neovascular glaucoma as the underlying cause. The patient received management for DKA in the ER and subsequent medicine ward. Various interventions were performed for glaucoma in the right eye, including addressing cataracts, which ultimately resulted in TASS. The patient was successfully treated with cryo-diode laser therapy.

## Introduction

Diabetes often presents in the emergency room (ER) with acute complications such as diabetic ketoacidosis (DKA) or hyperosmolar hyperglycemic nonketotic syndrome (HHNS). Type 1 diabetes tends to present with DKA. DKA is characterized by abdominal pain, which may mimic an acute abdomen, "fruity" breath odor or acetone breath, Kussmaul respiration (rapid, deep breathing), polydipsia, polyuria, polyphagia, and an altered state of consciousness. Ophthalmic complications caused by diabetes are typically discussed in the context of chronic effects. However, the case under discussion involves a patient presenting with prominent ophthalmic features, highlighting an uncommon manifestation of a commonly presenting disease entity.

The patient in question was also diagnosed with neovascular glaucoma (NVG), which involves the left eye more than the right eye. NVG is a rare type of glaucoma, with an incidence rate of approximately 6.6 per 10,000 [1]. While various pathological processes have been associated with NVG, diabetes is considered one of the primary causative factors [2]. It can lead to conditions like proliferative diabetic retinopathy (PDR), which serves as the underlying pathogenesis for NVG. Different interventions, ranging from intravitreal bevacizumab to pan-retinal photocoagulation, have been employed for the treatment of NVG [3].

The case further exhibited intravitreal bevacizumab-induced toxic anterior segment syndrome (TASS) during treatment. In ophthalmology, this anti-VEGF drug is used for various purposes, including neovascular age-related macular degeneration, retinopathy of prematurity, diabetic proliferative retinopathy, and diabetic macular edema [4,5]. While there are several complications associated with the use of intravitreal anti-VEGF drugs, TASS is a rare occurrence [6], with an incidence rate ranging from 0.014% to 0.082% [7]. This case report illustrates an unusual combination of different rare clinical ophthalmological pathologies, all within the context of type 1 diabetes mellitus (DM).

## Case presentation

A 20-year-old female presented to the emergency department with complaints of vomiting, fever, and severe bilateral eye pain lasting for six hours. The patient had multiple episodes of non-projectile, foul-smelling, watery, and yellow-colored vomiting. She had a known history of type 1 DM and glaucoma for four years. On examination, the patient appeared dehydrated and had a characteristic acetone breath. A Glasgow Coma Scale (GCS) score of 9 was recorded upon presentation. The left eye exhibited complete opacification in the corneal region. Signs of dehydration were observed during the general physical examination, and the patient exhibited Kussmaul respiration. These features collectively pointed toward a diagnosis of DKA.

Investigations conducted based on the features extracted from the patient's history and general physical examination were in line with DKA. The serum glucose level was measured at 607 mg/dL, the blood pH at 7.1, and the serum bicarbonate at 12 mEq/L. The serum tested positive for ketones, confirming the diagnosis of DKA and prompting emergency treatment.

After initial treatment, the patient's GCS was restored to 15. Although the complaint of bilateral eye pain remained moderate in intensity, it did not respond to painkillers. The ophthalmology department performed a fundoscopy on the right eye, which revealed full disc cupping. However, the examination of the left eye was hindered by corneal opacity. These initial findings suggested a diagnosis of glaucoma. A multidisciplinary approach led to the transfer of the patient from the medical ward to the ophthalmology ward after the initial management of DKA.

**Figure 1:**
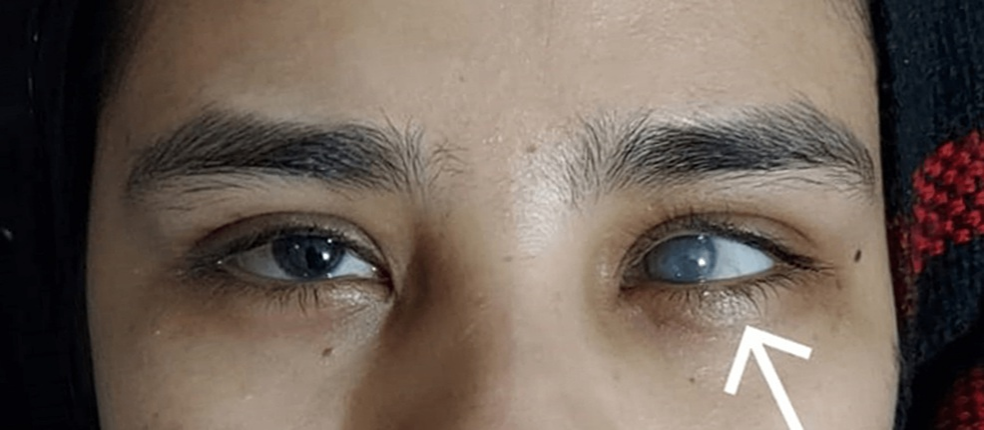
Left eye corneal opacification

Upon obtaining a detailed history, the patient revealed that she was diagnosed with glaucoma four years ago. Initially, she experienced severe eye pain and blurry vision in both eyes, more prominently in the left eye. Surgical intervention was performed as the initial treatment for glaucoma, which alleviated the symptoms at that time. However, four months later, the same symptoms reappeared in the left eye. A gonioscopy of the left eye indicated angle closure with neovascularization in all quadrants, while the right eye showed incomplete obliteration of the angle with neovascularization in one quadrant. A diagnosis of angle-closure neovascular glaucoma was established. The patient declined further surgical intervention and opted for medical treatment with timolol and brinzolamide eye drops, along with oral carbonic anhydrase inhibitors. It should be noted that the patient's blood sugar levels remained elevated throughout the entire duration due to non-compliance. Over time, the visual acuity in the left eye deteriorated, and the patient was eventually labeled legally blind. Left corneal vascularization had also developed by that point. Figure 1 shows the left corneal opacification.

At the time of presentation, the patient exhibited symptoms of epiphora, redness, and pain in the right eye. Applanation tonometry indicated a pressure of 40 mmHg in the right eye and 36 mmHg in the left eye. Examination revealed corneal edema, a bullous appearance, and a mid-dilated pupil in the right eye. Visual acuity at this stage was 3/60. Fundoscopic examination showed hard exudates in all quadrants of the retina, neovascularization on the disc, and dot-blot hemorrhage. Gonioscopy examination indicated obliteration of the angle in all four quadrants of the right eye, with neovascularization of both the iris and angle. A diagnosis of refractory closed-angle neovascular glaucoma was confirmed for the current symptoms. Initial treatment with intravenous mannitol, oral acetazolamide, and potassium chloride tablets was initiated, and cyclo-diode laser therapy was planned as a definitive treatment. Also, topical carbonic anhydrase and steroids were started.

**Figure 2:**
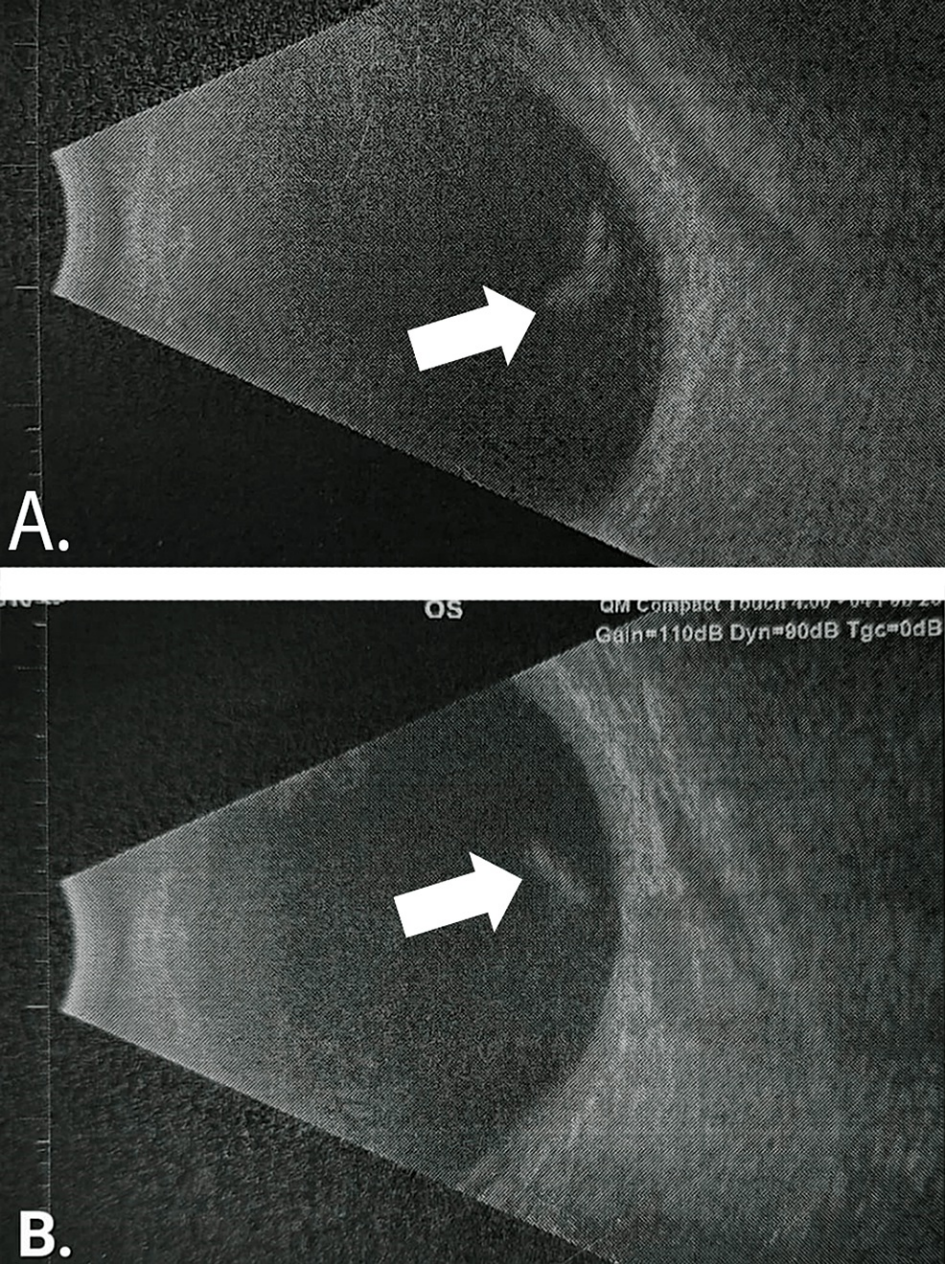
B-scan of both eyes (A) B-scan of the right eye and (B) shows a B-scan of the left eye. Arrows show minimal intragel hemorrhage. The retina and choroid are normal.

However, the initiation of cyclodiode laser therapy had to be delayed due to lens opacification in the right eye. Phacoemulsification with intraocular lens (IOL) implantation, accompanied by diode laser treatment, was planned for the patient. Before the procedure, an intravitreal bevacizumab 1.25 mg/0.05 mL injection was administered, which was taken from a new vial. Unfortunately, this intravitreal injection led to the emergence of new symptoms in the right eye, including corneal edema extending from limbus to limbus, ocular pain, and photophobia. This constellation of symptoms was identified as anti-VEGF-induced TASS. The diagnosis was supported by negative anterior segment bacterial culture results and B-scan imaging, which showed no involvement of the retina, choroid, or vitreous humor. The B-scan is elaborated below (Figure 2). Treatment with topical 1% prednisolone acetate was initiated, and cataract surgery was postponed for the duration of symptom resolution. Symptoms improved after three weeks, and the patient was cleared for cataract surgery on the left eye after four weeks.

The biometry of the right eye revealed a requirement for an IOL with a power of 27D. Consequently, phacoemulsification with IOL implantation under general anesthesia was performed on the right eye. The patient was prescribed extensive topical steroid use along with anti-glaucoma medications. Pan-retinal photocoagulation was performed on the right eye a week later to address features of proliferative diabetic retinopathy. While cryo-cycloablation was initially considered for glaucoma, cyclo-diode laser therapy was chosen due to the recurrent nature of the condition. The first session was conducted three weeks after cataract surgery, with the subsequent session scheduled three weeks later.

After one month following the second session, the patient presented with a best-corrected visual acuity (BCVA) of 20/200 in the right eye and an optimum intraocular pressure of 15 mmHg. The patient was advised to maintain good glycemic control and was scheduled for regular ophthalmic consultations.

## Discussion

Neovascular glaucoma (NVG) is a type of secondary glaucoma caused primarily by retinal ischemia and often associated with proliferative diabetic retinopathy (PDR) and central retinal vein occlusion (CRVO) [1]. Other causes can include tumors, inflammatory diseases, and systemic vascular disorders. Treatment for NVG typically involves a combination of medical and surgical interventions.

Medical therapy for NVG includes the use of aqueous suppressants, prostaglandins, osmotic agents, cycloplegics, and steroids. Topical or systemic use of these depends on the specific condition being treated and its severity. Surgical treatment options vary, with trabeculectomy offering lower intraocular pressure but a higher risk of conjunctival scarring, hence not generally considered for treatment of NVG, while aqueous shunts are indicated in cases of active neovascularization to reduce the risk of intraoperative hemorrhage. Intraoperative use of anti-VEGF agents has shown promise in decreasing hemorrhage risk and improving prognosis [3,8].

In the discussed case, the lack of surgical intervention and poor control of comorbidities resulted in a poor prognosis. This highlights the importance of a combination of medical and appropriate surgical interventions for favorable long-term outcomes.

Toxic anterior segment syndrome (TASS) is characterized by severe and acute intraocular inflammation following surgery, resembling sterile endophthalmitis. Symptoms typically appear within 12-24 hours after surgery [9]. TASS can be caused by various factors, including bacterial endotoxins, topical ointments, intraocular medications such as anti-VEGF agents, and denatured ophthalmic viscosurgical devices [10]. The diagnosis is based on symptoms, a slit lamp examination, a dilated fundus examination, and sometimes an ultrasound B-scan. Treatment involves the use of topical and systemic steroids based on the severity of inflammation, with surgical intervention rarely necessary.

Anti-VEGF-induced TASS can occur as an immune-mediated response to bevacizumab following intravitreal injection or due to breakdown products resulting from faulty storage [11]. The exact cause of bevacizumab-induced TASS remains unclear. Treatment includes the use of topical steroids and prophylactic antibiotics [12]. However, intervention with vitrectomy is reserved for severe cases [6].

Differential diagnoses for TASS include infectious endophthalmitis and uveitis [13]. While for NVG, they include retinal detachment, intraocular tumors, and uveitis. The presentation of two clinically rare conditions, acute angle closure neovascular glaucoma and anti-VEGF-induced TASS, along with uncontrolled type 1 diabetes mellitus, posed significant diagnostic and interventional challenges for the medical professionals involved. Timely and appropriate interventions could have potentially improved the overall prognosis.

## Conclusions

This case report highlights the uncommon manifestations of a commonly encountered condition, diabetic ketoacidosis, as well as the rare occurrence of neovascular glaucoma and bevacizumab-induced TASS. These series of events exemplify Murphy's Law, where everything that could have gone wrong did go wrong. The report contributes to the field by providing valuable insights into the pathophysiology of bevacizumab-induced TASS and adding to the existing biostatistics on this condition.
